# Factors Associated With the Use of the Contraceptive Implant Among Women Attending a Primary Health Clinic in Cape Town, South Africa

**DOI:** 10.3389/fgwh.2021.672365

**Published:** 2021-08-06

**Authors:** Kim Jonas, Mikateko Mazinu, Moira Kalichman, Seth Kalichman, Carl Lombard, Chelsea Morroni, Catherine Mathews

**Affiliations:** ^1^Health Systems Research Unit, South African Medical Research Council, University of Cape Town, Cape Town, South Africa; ^2^Adolescent Health Research Unit, Department of Psychiatry and Mental Health, University of Cape Town, Cape Town, South Africa; ^3^Biostatistics Research Unit, South African Medical Research Council, University of Cape Town, Cape Town, South Africa; ^4^Department of Psychology, University of Connecticut, Storrs, CT, United States; ^5^Centre for Reproductive Health, University of Edinburgh, Edingburgh, United Kingdom; ^6^Women's Health Research Unit, School of Public Health and Family Medicine, University of Cape Town, Cape Town, South Africa; ^7^Botswana Harvard Acquired Immune Deficiency Syndrome Institute Partnership, Gaborone, Botswana

**Keywords:** long-acting reversible contraceptives, subdermal contraceptive implant, contraception, family planning, South Africa

## Abstract

**Background:** Long-acting reversible contraceptives (LARCs), which include the subdermal contraceptive implant and intrauterine contraception, offer women safe, highly effective, long-term pregnancy prevention, and have expanded contraceptive options. The implant greatly expands LARC options for South African women as it is available free of charge at public health facilities, but little is known about factors associated with its uptake. This study describes factors associated with the intention to use the implant, including knowledge and beliefs about the implant and perceived outcome expectancies of implant use among women in Cape Town, South Africa.

**Methods:** Between 2015 and 2016, the authors conducted a quantitative, cross-sectional survey among adult women attending a public, primary health clinic in Cape Town, South Africa. Using a structured questionnaire, they measured knowledge, awareness, and attitudes, perceived outcome expectancy, and the intention to use the contraceptive implant in future among the women.

**Results:** The authors surveyed 481 women (mean age 29.1 years). Most of the participants (*n* = 364, 75.6%) had heard about the implant, 45 (9.4%) were currently using it, and 97 (20.2%) intended to use it in the future. Knowledge about the safety of the implant, beliefs about its effectiveness, and the ease of insertion and removal, and support from intimate partners were positively associated with the current use and intentions to use the implant in the future.

**Conclusions:** Limited knowledge of the implant, having completed secondary schooling, support from partner for women to use implant and the perceived outcome expectancies of using the implant were factors significantly associated with the intention to use the implant. Ensuring that the contraception information is available in all South African languages, regardless of education levels in women, and that comprehensive contraception education and counseling is provided during all family planning might help improve the uptake of contraceptives, including the use of the implant in the country.

## Background

Long-acting reversible contraceptives (LARCs), which include the subdermal contraceptive implant and intrauterine contraception (IUC), offer women safe, highly effective, long-term pregnancy prevention, and expanded contraceptive options. The South African Department of Health (DoH) 2012 policy and guidelines emphasized the importance of expanding contraceptive choice, and in particular increasing access to the LARC options (1). The implant, which has been widely available at no cost to clients in South African public sector clinics, since its introduction in 2014 was part of the implementation of these policies, and greatly expanded LARC options for South African women (1). LARCs are the most effective methods of contraception available with considerably lower unintended pregnancy rates compared to the shorter-acting contraceptives, such as oral contraceptive pills or injectables because of their long duration of contraceptive effect and because regular adherence is not required by the user (2–4). The use of LARCs has been shown to reduce unintended pregnancies among women in general, reduce repeat pregnancies among adolescents, and reduce repeat medically induced abortions (5–7). Studies of the cost-effectiveness of different contraceptive methods in high-income countries (HICs) have favored the use of LARC methods (2, 3, 8–10). However, promotion of any specific method of contraception, including LARCs, as effective as they are, has been cautioned by reproductive health and rights advocates as it undermines the reproductive autonomy of women.

The US-based National Women's Health Network (NWHN) argues that we should not only fight for improving access to contraception and abortion care services but also fight for reproductive autonomy and eliminate coercive behaviors in family planning programs and practices (11). This is in line with the National Integrated Sexual and Reproductive Health and Rights (SRHR) Policy of South Africa, which addresses cross-cutting issues relating to sexual and reproductive health (SRH) service provision that underpin quality, comprehensive, and integrated SRHR service provision in the country (12). However, barriers to contraception access persist in the country, and the implant is no exception. Despite being freely available and more effective, there has been low uptake of the implant among South African women (13, 14). Studies from different settings around the world have identified factors attributed to low uptake of LARCs, such as limited knowledge and training of healthcare providers with LARC methods, method cost, and the limited number of healthcare providers trained to provide LARCs (6, 7, 14–16). Globally, factors attributed to the low uptake of LARCs among women themselves include the limited knowledge and awareness of the LARC methods, and cost (5, 17–19). In South Africa, limited awareness and knowledge of the implant, and awareness of other different contraceptive methods, have been identified as the attributing factors to the low uptake (14). Additionally, it is reported that myths and misconceptions about the side effects and risks associated with the LARC methods exist among South African women (6, 7, 20).

Few studies, however, have examined the factors associated with the use of, and intentions, to use the implant in the future among women in South Africa. It has been reported that most women use the implant because of its convenience as no user action required for several years after insertion, whereas side effects, in particular altered bleeding patterns, have been reported as the main reason for discontinuation (13). Furthermore, insufficient pre-counseling on implant side effects is thought to contribute to method discontinuation in South Africa and elsewhere in the world (13, 14, 21, 22). The aim of this study, therefore, was to describe the factors associated with the intention to use the implant in the future and investigate the perceived outcome expectancies of using the implant among women of reproductive age attending a primary health clinic in Cape Town. Outcome expectancies are defined as anticipated consequences, positive or negative consequences resulting from doing something (23). We investigated the intention to use the implant to assess whether women have any consideration of ever using the implant in the future, given the effort and investment made by the South African government in making this typically costly method of contraception freely available. We also investigated perceived outcome expectancies for using a contraceptive implant to understand what would make women want to use the implant or not in the future. This information may help family planning programs to better understand the low uptake of the implant and where to direct their efforts on improving the uptake of the implant.

## Methods

### Research Design and Study Setting

This cross-sectional survey was conducted among adult women attending a primary health clinic in Cape Town, South Africa. The clinic was selected because it is a typical public clinic in a poor, peri-urban setting. It offered a range of public primary health services with a primary focus on sexually transmitted infection (STI), HIV, and TB, and also SRH services.

### Participant Sampling

We invited all women age 18 years and older who were receiving family planning and STI services at the clinic between February 2015 and February 2016 to complete a brief anonymous survey while waiting to see the clinic nurse. To be eligible to participate, women had to be able to read and write in English or isiXhosa; and women who were able to give consent. As this was a specific clinic for family and STI treatment, 500 women were eligible and were invited to participate, with 481 completing the survey. Those who declined to participate cited time constraints.

### Data Collection Tool

We used a paper–pencil questionnaire format, requiring approximately 10 min for completion, which participants self-completed in their preferred language (either English or Xhosa). Because of the sensitive nature of the questions, we offered self-administered survey completion to give participants privacy to answer the questions as truthfully as possible. Demographic questions included questions regarding their relationship status. Main sexual partner was defined as a sexual partner one has for 6 or more months and included husbands (for those who are married), whereas, casual partner was defined as a “secret” sexual partner one has sex with occasionally and included the one-night-stand sex partners. In the South African context, a casual partner is known as “*umakhwapheni*” and the term is used to describe any person whom a man or woman has sex with occasionally with their sexual relationship kept “secret” from others. For this study, we translated the commonly used term for a casual partner (*umakhwapheni*) into “secret” sex partner, which participants are used to and can relate to. We included reproductive health questions, such as number of pregnancies and reproductive intentions.

We included questions on ever and current use of contraceptives, and awareness and knowledge of, and attitudes toward the contraceptive implant, in particular. To assess ever use of contraceptives, women were asked the following question: “have you ever used any method of modern contraception?” (see Table 1). We asked about knowledge of different types of contraceptive methods, the sources of information about the methods, and whether a health worker had ever offered them the method.

**Table 1:** Cross-tabulation of demographic characteristics, reproductive intentions, and contraception use by intention to use the contraceptive implant in the future among women in Cape Town, South Africa.

	**Intend to use contraceptive implant in future?**				
**Variables**	**Yes *n* (%)^#^**	**Unsure *n* (%)**	**No *n* (%)**	**Total *n***	***p*-value (Chi squared)**
Total	97 (21)	131 (28)	235 (51)	463	
**Demographics**					
Age					
<24 years	28 (20)	37 (27)	73 (53)	138	0.83
>24 years	69 (21)	94 (29)	162 (50)	325	
Employed					
Yes	30 (18)	50 (30)	84 (51)	164	0.50
No	67 (22)	80 (27)	151 (51)	298	
Education					
Completed high school	44 (29)	44 (29)	64 (42)	152	**0.01**
Did not complete high school	53 (17)	85 (28)	171 (55)	309	
Main sexual partner					
Yes	78 (20)	109 (29)	196 (51)	383	0.86
No	17 (23)	22 (29)	36 (48)	75	
Casual sexual partners					
Yes	20 (29)	20 (29)	28 (42)	68	0.09
No	72 (19)	110 (28)	204 (53)	386	
Marital status					
Yes	14 (21)	19 (28)	34 (51)	67	1.00
No	82 (21)	110 (28)	199 (51)	391	
Living with sexual partner or husband					
Yes	31 (21)	47 (31)	72 (48)	150	0.60
No	66 (21)	84 (27)	162 (52)	312	
**Reproductive intentions and contraceptive use**					
Ever been pregnant					
Yes	70 (21)	85 (26)	176 (53)	331	0.12
No	26 (20)	46 (35)	59 (45)	131	
Currently pregnant					
Yes	5 (28)	4 (22)	9 (50)	18	0.34
No	89 (21)	116 (28)	211 (51)	416	
Unsure	2 (7)	11 (41)	14 (52)	27	
When do you intend to have a(nother) child					
>36/unsure/don't want a child	82 (20)	115 (28)	207 (52)	404	0.65
In the next 12–36 months	14 (26)	15 (27)	26 (47)	55	
HIV status					
Positive	28 (27)	29 (28)	46 (45)	103	0.19
Negative	62 (19)	86 (27)	171 (54)	319	
Unsure/refuse to answer	7 (17)	16 (39)	18 (44)	41	
Main sexual partner or husband want child					
No	61 (22)	75 (28)	134 (50)	270	0.64
Yes	36 (19)	56 (29)	98 (51)	190	
Ever used any modern family planning method^$^					
Yes	77 (22)	96 (27)	179 (51)	352	0.56
No	20 (18)	35 (32)	55 (50)	110	
Partner's feelings about contraceptive use					
Supportive	56 (20)	74 (26)	157 (54)	287	0.10
Other^*^	40 (23)	56 (32)	77 (45)	173	
Ever had implant					
Yes	28 (43)	9 (14)	28 (43)	65	**0.01**
No	69 (17)	122 (31)	206 (52)	397	
HCW ever offered implant					
Yes	57 (30)	40 (21)	95 (49)	192	**0.01**
No	40 (14)	91 (34)	140 (52)	271	
Currently have implant					
Yes	22 (41)	6 (11)	26 (48)	54	**0.01**
No	75 (18)	124 (30)	208 (52)	407	

* *Not supportive, neutral/don't know, HCW, healthcare worker*.

# *Row percentages rounded to add up to 100%*.

$ *Modern family planning method was defined as injectable, birth control pill, and LARC methods. Bold value indicates significant*.

To assess knowledge of the implant, participants were provided with a set of seven statements regarding the safety (e.g., “Most women can safely use the implant”) and effectiveness of the implant (e.g., “The implant is very effective at preventing pregnancy”) (see Table 2). A three-point Likert scale was used (agree, disagree, and unsure). The internal consistency of the knowledge composite was good, given that our intent was to assess a broad range of information and misinformation about the implant (Cronbach's α: 0.64; 95% CI: 0.60–0.69).

**Table 2:** Ordinal regression and cross-tabulations of knowledge and perceive outcome expectancies of the contraceptive implant with the intention to use the implant in the future among women in Cape Town, South Africa.

	**Intention to use contraceptive implant in future**					
**Variables**	**Yes *n* (%)**	**Unsure *n* (%)**	**No *n* (%)**	**Total**	**OR (95% CI)**	***P*-value**
**Knowledge**						
Most women can safely use the implant						
Disagree (ref)	19 (23)	19 (23)	44 (54)	82	**2.17 (1.66–2.87)**	**0.01**
Unsure	26 (12)	75 (35)	116 (53)	217		
Agree	51 (31)	37 (23)	74 (46)	162		
Teenagers can safely use the implant						
Disagree (ref)	9 (16)	9 (16)	37 (67)	55	**1.76 (1.38–2.25)**	**0.01**
Unsure	19 (10)	69 (36)	105 (54)	193		
Agree	69 (32)	53 (25)	93 (43)	215		
Women who have not yet had a baby can safely use the implant						
Disagree (ref)	12 (22)	14 (25)	29 (53)	55	**1.30 (1.05–1.63)**	**0.02**
Unsure	13 (11)	51 (44)	53 (45)	117		
Agree	72 (25)	66 (23)	150 (52)	288		
Women with HIV can safely use the implant						
Disagree (ref)	5 (12)	11 (27)	25 (61)	41	**1.34 (1.05–1.72)**	**0.02**
Unsure	8 (8)	36 (35)	60 (58)	104		
Agree	83 (26)	84 (27)	149 (47)	316		
The implant is very effective at preventing pregnancy						
Disagree (ref)	39 (29)	30 (23)	64 (48)	133	**1.89 (1.44–2.51)**	**0.01**
Unsure	33 (14)	79 (33)	129 (54)	241		
Agree	24 (28)	21 (24)	41 (48)	86		
The implant protects against STIs and HIV						
Disagree (ref)	45 (27)	32 (19)	90 (54)	167	0.92 (0.70**–**1.21)	0.56
Unsure	33 (15)	80 (37)	105 (48)	218		
Agree	19 (25)	18 (24)	39 (51)	76		
**Perceived outcome expectancies [I think that if I were to use the implant…]**						
It could be easily inserted						
Disagree (ref)	19 (23)	19 (23)	44 (54)	82	**1.31 (1.01–1.68)**	**0.04**
Unsure	26 (12)	75 (35)	116 (53)	217		
Agree	51 (31)	37 (23)	74 (46)	162		
It could be easily removed						
Disagree (ref)	9 (16)	9 (16)	37 (67)	55	**1.82 (1.39–2.40)**	**0.01**
Unsure	19 (10)	69 (36)	105 (54)	193		
Agree	69 (32)	53 (25)	93 (43)	215		
It will be very convenient						
Disagree (ref)	12 (22)	14 (25)	29 (53)	55	1.06 (0.82**–**1.36)	0.66
Unsure	13 (11)	51 (44)	53 (45)	117		
Agree	72 (25)	66 (23)	150 (52)	288		
It would be very good, because it lasts long						
Disagree (ref)	5 (12)	11 (27)	25 (61)	41	**1.54 (1.17–2.05)**	**0.00**
Unsure	8 (8)	36 (35)	60 (58)	104		
Agree	83 (26)	84 (27)	149 (47)	316		
I'd worry about gaining weight						
Disagree (ref)	39 (29)	30 (23)	64 (48)	133	0.95 (0.73**–**1.23)	0.71
Unsure	33 (14)	79 (33)	129 (54)	241		
Agree	24 (28)	21 (24)	41 (48)	86		
I'd worry about bleeding changes						
Disagree (ref)	45 (27)	32 (19)	90 (54)	167	1.04 (0.80**–**1.34)	0.78
Unsure	33 (15)	80 (37)	105 (48)	218		
Agree	19 (25)	18 (24)	39 (51)	76		
I could get pregnant soon after it's been removed						
Disagree (ref)	28 (34)	18 (22)	36 (44)	82	1.05 (0.81**–**1.35)	0.74
Unsure	28 (12)	74 (31)	133 (57)	235		
Agree	41 (28)	39 (27)	66 (45)	146		
It could stop me from getting pregnant and make it harder in future						
Disagree (ref)	29 (24)	28 (24)	62 (52)	119	1.16 (0.89**–**1.51)	0.27
Unsure	40 (16)	81 (32)	133 (52)	254		
Agree	28 (31)	21 (24)	40 (45)	89		
It could move around my body						
Disagree (ref)	21 (25)	27 (32)	37 (44)	85	1.01 (0.79**–**1.31)	0.91
Unsure	32 (14)	75 (33)	123 (53)	230		
Agree	44 (30)	29 (20)	75 (51)	148		
It would be painful						
Disagree (ref)	30 (22)	35 (25)	73 (53)	138	1.20 (0.94**–**1.53)	0.14
Unsure	29 (14)	73 (35)	107 (51)	209		
Agree	38 (33)	23 (20)	55 (47)	116		
It could harm future babies						
Disagree (ref)	12 (24)	8 (16)	29 (59)	49	1.28 (0.97**–**1.70)	0.08
Unsure	34 (14)	88 (36)	121 (50)	243		
Agree	51 (30)	35 (20)	85 (50)	171		

*P is significant at p ≤ 0.05. Bold value indicates significant*.

To measure perceived outcome expectancies of the use of the contraceptive implant, participants were asked to imagine how it would be for them if they were to use the implant (see Table 2). The women were provided with 11 statements, each reflecting a positive and a negative outcome expectancy [e.g., “I think that if I were to use the implant it would be very good because it lasts for a long time” (positive) and “I think that if I were to use the implant, the implant would move around my body” (negative)]. A three-point Likert scale was used (agree, disagree, or unsure). The items of this composite were used as specific attitudinal indicators of perceived outcome expectancies of personal use. Finally, to measure the intention, participants were asked whether they thought they might use the contraceptive implant in the future (response options: yes, no, or unsure). We measured the intention to assess whether women have any future plans of using the implant as a preferred long-term method of contraception (Tables 1, 2). A copy of the questionnaire with the full statements is provided as an additional file [Supplementary Material 1: Questionnaire].

### Procedure

A research assistant approached the women who were attending and receiving family planning and STI services at the clinic and briefly informed them about the study. Those interested were then screened for eligibility and enrolled upon meeting the eligibility criteria described above. The research assistant conducted the informed consent process with interested participants in their preferred language, which was mainly isiXhosa. Participants were then given a copy of their signed consent form with an information sheet containing details of the principal investigator, ethics committee member, and the study coordinator whom they contact if they need further information about the study or they want to report any misconduct about the study procedures. Participants were given the paper survey on a clipboard, and they sat at some distance from others in the waiting room while they completed the survey. The survey was translated into isiXhosa, a native language spoken by the majority in the community surrounding the clinic. An instruction was provided before every question (which was also translated) to help participants understand how to respond to the questions. They used a paper folder to shield the survey they were completing from the view of others. This is not ideal, and we have acknowledged this as another limitation of this study.

The study was approved by the South African Medical Research Council's Ethics Committee (EC018-10/2013) and the University of Connecticut Institutional Review Board (H12-340).

### Data Analyses

Data were analyzed using SPSS version 25 (IBM Corp, Armonk, New York) and the R Program version 3.0 (R Core Team, Austria). Prior to analysis, items were rescored so that the valid statements and positive attitudes scored the highest. A score was composed of the seven items of knowledge composite and used in the multivariate analysis. We did not compose a score for the perceived outcome expectancy composite, as we used each item as a specific indicator of outcome expectancies.

Descriptive statistics were generated to get an overview of the sample characteristics. Univariate ordinal regression was then conducted to determine the associations between knowledge, and perceived outcome expectancies, and the intention to use the contraceptive implant in the future. Multiple ordinal regression was performed to examine the factors independently associated with the intention of women to use the contraceptive implant in the future. The regression model was fitted under the assumption of proportional risk. All variables that were significant in the univariate analysis together with the knowledge scale score were included in the multiple regression analysis to further explore the associations. The variables were entered in a stepwise approach, both in the univariate and multivariate analyses with complete case analysis applied. The level of significance for the statistical tests in the study was set at *p* ≤ 0.05). The proportion of missingness in this study was <4% and occurred at an item level. According to Schafer (24), a missing rate of 5% or less is considered not significant. Thus, no missing data mechanism was used to impute the missingness.

## Results

### Description of Participants

A total of 481 women completed the survey, representing an approximately 80% response rate. The age of participants ranged from 18 to 48 years, with the mean age of 29.1 years and SD of 6.8, and with 28.7% (*n* = 138) below the age of 24 years. About 34% of participants were employed. The majority (64.2%, *n* = 309) of participants did not complete secondary schooling. About 79.6% (*n* = 383) of participants reported having a main sexual partner (sex partner whom one has sex with for 6 months or more) and 15.6% (*n* = 75) reported having a casual sexual partner. A casual sex partner, also known as a “secret” sex partner, is a partner whom one has sex with occasionally. About 14% of participants were married. Among women who reported having a main partner and those that reported being married, 31.2% were living with the partner. About 71% of the participants had ever been pregnant before, and 3.7% (*n* = 18) were currently pregnant. About 12.5% (*n* = 55) of the participants reported intending to have a child or become pregnant in the next 1 to 3 years. With regard to HIV status, 21.4% (*n* = 103) of the women reported being HIV positive, whereas 8.5% (*n* = 41) were either unsure or declined to answer.

### Contraceptive Use and Intentions

Nearly all women (99.8%) reported having ever used a modern contraceptive method defined as injectable, oral contraceptive pill, and the LARCs. Among the women who ever used modern contraception, injectable contraception was reported as the most common method ever used at 90.1% followed by the oral contraceptive at 27.0%. Ever use of LARCs was lower, with the implant at 11.6% and the intrauterine device at 10.8% among those who had used a method. With regard to condom use, among those who ever had sex, 77.8% have used a male condom and 16.3% have used a female condom.

With the current use of contraceptives, most women (71.1%) reported currently using the injectable, followed by the oral contraceptive at 15.8%. About 9.4% of women were currently using the implant, and 4.2% were using an intrauterine device (IUD). Condoms were currently used by 57.8% of all the women in this study. Only 9.2% of all women were currently not using any method of contraception. Among all participants, 75.6% have heard about the contraceptive implant and 21.0% intended to use the implant in the future, and 28.3% were unsure whether they would use it, and 51.0% did not intend to use it.

Table 1 presents the demographic characteristics, reproductive intentions, and contraceptive use by the intention to use the implant among participants in the future. Intentions to use the implant were positively and significantly associated with having completed high school, having used the implant before, ever having been offered the implant by a health worker, and currently having the implant (see Table 1).

### Factors Associated With the Intention to Use a Contraceptive Implant

Table 2 presents a univariate analysis between the items in the knowledge and the perceived outcome expectancy composites, against the intention to use the implant among all women in the study. All variables under the knowledge composite were significantly associated with the intention to use the contraceptive implant (see Table 2). Women who had better knowledge about the safety of the implant were more likely to intend to use it than those with poor safety knowledge of the implant. Similarly, women who had better knowledge about the safety of implants for use by teenage girls, and for women infected with HIV were more likely to intend to use it. With regard to perceived outcome expectancy composite, women who believed that using the contraceptive implant is good because it lasts longer, the implant can be easily removed, and it can be easily inserted were more likely to have the intention to use the implant.

The multiple ordinal regression analysis (Table 3) shows that women who had completed high school were more likely to intend to use the implant than women who had not (OR: 2.04; 95% CI: 1.35–3.11, *p* < 0.01). Women who had ever been pregnant (OR: 1. 62; 95% CI: 1.00–2.62, *p* = 0.05), and women who felt they had the support of their partner to use contraception (OR: 1.51, 95% CI: 1.01–2.27, *p* = 0.05) were more likely to intend to use the implant compared to women who had not been pregnant and women whose partners were not supportive of contraception use, respectively. Participants who thought that the implant could easily be removed were more likely to intend to use it than those who did not believe it could be (OR: 1.59, 95% CI: 1.15–2.23, *p* = 0.01).

**Table 3:** Multiple ordinal regression of intent to use contraceptive implant in future on selected socio-demographic, knowledge, and perceived outcome expectancy variables among women in Cape Town, South Africa.

		**Intention to use contraceptive**			
		**implant in future**			
**Variables**		**OR**	**95% CI**		***p*-value**
**Demographics**					
Age	(≤ 24^*^ vs. >24)	1.49	0.92	2.44	0.11
Living with sexual partner or husband	(Yes vs. No^*^)	1.21	0.79	1.83	0.42
Education	(Completed high school^*^ vs. not completed)	**2.04**	**1.35**	**3.11**	** < 0.01**
**Childbearing, fertility intensions, and contraception**					
Previous pregnancy	(Yes^*^ vs. No)	1.62	1.00	2.62	0.05
When do you intend to have a(nother) child	(<36 vs. >36 months)	1.21	0.66	2.21	0.46
Husband want child	(Yes vs. no^*^)	0.75	0.50	1.10	0.11
Clinic visit for family planning	(Yes vs. No^*^)	0.81	0.53	1.24	0.26
Partner's feeling about contraceptive use	(Supportive^*^ vs. other)	**1.51**	**1.01**	**2.27**	**0.05**
HCW ever offered implant	(Yes vs. No^*^)	1.14	0.76	1.71	0.39
Ever had implant	(Yes vs. No^*^)	1.70	0.91	3.16	0.07
**Knowledge**					
Knowledge about the implant	(composite score)	1.70	0.91	3.16	0.07
**Perceived outcome expectancies [I think that if I were to use the implant…]**					
It could be easy to remove	(Disagree^*^ vs. unsure vs. agree)	**1.59**	**1.15**	**2.23**	**0.01**
I could get pregnant soon after removing it	(Disagree^*^ vs. unsure vs. agree)	1.60	1.16	2.22	0.40
It could harm future babies	(Disagree^*^ vs. unsure vs. agree)	1.03	0.74	1.45	0.12

* *Reference category, Bold = significant*.

## Discussion

This study examined factors associated with the intention to use the contraceptive implant, including knowledge and beliefs about the implant and perceived outcome expectancies of implant use among women of reproductive age in a primary health clinic in Cape Town, South Africa. The findings indicate that the intention to use the contraceptive implant is significantly associated with the correct knowledge about the implant, having completed secondary schooling, having been pregnant before, support of partner for women to use an implant, and the perceived outcome expectancies of using the implant. These findings are not surprising as limited knowledge of the different contraceptive methods in general, not only of the implant but also low levels of education and lack of partner support to use contraceptives have been reported in a number of studies, both locally and globally (5, 17–19, 25–29). Limited knowledge and awareness of the implant, in particular among women in the country, is thought to contribute to its low uptake (14). The majority of the participants in this study did not complete secondary school education, and this finding is similar to other research in the country where a substantial proportion of women with an unmet need for contraception were those with low levels of education and not in professional employment (26).

Given that most women know more about injectable and oral contraceptives and are more likely to use these methods of contraception, it is likely that they may not be using the implant and other methods of contraception because they are not well-informed about them. It is also possible that women are not receiving comprehensive contraception education and counseling during their family planning visits, an opportunity to provide accurate and consistent information about all available methods of contraception to all women. Patel et al. (22) highlighted the need to educate women before providing them with the implant as its side effects, such as the change in bleeding pattern leads to early removal and discontinuation of the implant. A study conducted by Brown (21) in the UK also concluded that adequate counseling for women and adolescents about the side effects of the implant is extremely important to reduce the rate of discontinuation. Additionally, limited knowledge and awareness of the implant was associated with poor uptake by women, including adolescent girls in the US (27). It has also been highlighted in South Africa that education and counseling during family planning visits are limited, particularly on the effectiveness of the different methods available (13, 28). If women are not well-informed about the contraceptive implant and are unprepared for the side effects, then they have negative perceived outcome expectancies of the implant use and are unlikely to intend to use it, undermining efforts to improve accessibility and availability of the method. The limited knowledge of the implant could be improved by ensuring that all information related to contraceptives, including the implant, is available in the different South African languages where most women can easily access it regardless of their education level and ensure comprehensive contraception education and counseling during all family planning visits. Knowledge and awareness about the different methods of contraception, including the implant, can also help improve the perceived outcome expectancies, eliminate the misconceptions about them, particularly those who can have serious unintended consequences, such as increasing the rates of STIs among some implant users due to the belief that it prevents them. Therefore, tailored interventions to specifically meet the needs of all women, regardless of their level of education, should be prioritized when designing interventions to improve knowledge about, and attitudes toward the implant and other contraceptives, while increasing their intentions to use them.

Improved knowledge of contraceptive methods may improve the uptake of other methods, such as the uptake of the implant in the country. This is not to encourage the promotion of the implant or other LARC methods but to highlight the importance of comprehensive contraception counseling where all methods are discussed with all women seeking family planning to enable them to make informed decisions on the method most suitable for them. Furthermore, comprehensive contraception education and counseling will help align the specific reproductive needs and desires of an individual with the ideal contraceptive method of her choice. As stated in the 2012 contraception policy and guidelines, LARCs provide women with more contraceptive options in which they can choose based on their reproductive needs and intentions (1), and they must be viewed and offered in that way and not promoted over other methods as that compromises the women's reproductive rights. Therefore, family planning program and interventions in the country must place women at the center of the service and provide them with all relevant information about contraceptive methods available to them, in line with their reproductive needs and desires to ensure that no woman is steered or coerced to use any method of contraception.

The finding on partner support being associated with the intention to use the implant is not unfamiliar in the family planning services, and in South Africa as gender inequalities continue to disproportionately affect women. Pillay et al. (13) also reported on lack of partner support as another reason some women removed or discontinued the implant. In our previous study, we found that limited knowledge of contraceptives among men was the main factor contributing to lack of support of men for use of contraceptives of female intimate partners (29). Although, engaging men in family planning programs has received some attention recently, this finding highlights that more efforts are needed to alleviate gender inequalities and empower women to make their own reproductive choices and not fear for their partners in taking such decisions.

There are some important limitations to be considered in interpreting the findings of this study. The first limitation is the cross-sectional nature of the study, which cannot infer causality, i.e., improving the knowledge and attitude of women about the contraceptive implant will not necessarily result in the increase of the intention to use the implant, and the uptake thereof. The second limitation is the setting in which this study was undertaken, limiting the generalizability of study findings. The clinic was a family planning and STI treatment clinic only, and therefore, only women who were coming to seek family planning services and/or receiving STI treatment were included in the study. We acknowledge the limitations of privacy within this public facility in which we as researchers did not have control of, although, we did our best to ensure participants complete the questionnaire in the safest and most private way the facility could afford us. We also acknowledge that the intention to use a method is not a simple straightforward concept to understand, and the fact that the response is for the future in that it is a possibility rather than a certainty, is another limitation for this study. Despite these limitations, the findings of this study highlight the factors that need to be addressed in order to increase the knowledge and intention of women to use the contraceptive implant and ultimately improve the overall uptake of contraceptives among women in the country.

## Conclusions

The findings of this study show that knowledge and attitude play an important role in the intention to use any family planning method among women, but more so toward the implant. The support of the partner for contraceptive use is also an important factor for the intention of women to use contraceptives, and thus partners need to be included as well when planning and rolling out interventions to increase knowledge and awareness, and the intentions to use contraceptives including the LARCs. Our study highlights the importance of comprehensive contraception education and counseling provision during family planning visits to empower women to make informed decisions about contraceptives that best suit their needs and preferences. This may also aid in improving their overall knowledge and attitudes toward contraceptive methods and eliminates the existing myths and misconceptions around contraceptives. Provision of comprehensive contraception education and counseling during family planning consultation is an important step toward improving the knowledge and awareness about different contraceptive methods, including the implant among women, and subsequently the uptake thereof. These findings are valuable and offer a better understanding of the reasons behind the low uptake of, and the discontinuation of the contraceptive implant among women in the country, as the government has invested in the provision of the implant at no cost. More research is needed to explore ways in which contraceptive education can be incorporated in the school curriculum to improve knowledge and awareness of adolescent girls on various contraceptive methods and help improve their uptake. Efforts to train health providers in the provision of education and counseling about LARCs in general and encourage them to advocate for the LARCs first need to be escalated to improve the implant uptake among women in South Africa.

## Data Availability Statement

The raw data supporting the conclusions of this article will be made available by the authors, without undue reservation.

## Ethics Statement

The studies involving human participants were reviewed and approved by South African Medical Research Council's Ethics Committee (EC018-10/2013) and the University of Connecticut Institutional Review Board (H12-340). The patients/participants provided their written informed consent to participate in this study.

## Author Contributions

KJ and MM performed the data analysis and interpretation of the data. KJ prepared the first draft of the manuscript. CMo, MK, SK, CL, and CMa contributed to the analysis, interpretation of results, and writing of the manuscript. All authors participated in the conception and design of the study, participated in the reviewing of the content for submission, and approved the final version of the manuscript.

## Conflict of Interest

The authors declare that the research was conducted in the absence of any commercial or financial relationships that could be construed as a potential conflict of interest.

## Publisher's Note

All claims expressed in this article are solely those of the authors and do not necessarily represent those of their affiliated organizations, or those of the publisher, the editors and the reviewers. Any product that may be evaluated in this article, or claim that may be made by its manufacturer, is not guaranteed or endorsed by the publisher.

## Abbreviations

Cu IUD: copper intrauterine device
HIV: human immunodeficiency virus
HIC: high-income countries
IUD: intrauterine device
LARC: long-acting and reversible contraceptives
LMIC: low- and middle-income countries
STI: sexually transmitted infections
SPSS: Statistical Package for the Social Sciences
TB: tuberculosis.
