# Supplementary material for: Factors Associated With the Use of the Contraceptive Implant Among Women Attending a Primary Health Clinic in Cape Town, South Africa
Source: Front Glob Womens Health. 2021 Aug 6;2:672365. doi: 10.3389/fgwh.2021.672365 (PMC8594047; doi:10.3389/fgwh.2021.672365)
Supplement: Supplementary file 1 [file Data_Sheet_1.PDF]

______________ (1)

Entry 1: ____________

Entry 2: ____________

Cleaned ______________

Date: (2)	Staff (3)

IMBUMBA Survey 3-W

Ten Minutes of Your Time Can Help Improve Health Services

Thanks for taking time to fill out this survey.

This survey is completely anonymous.
Please do not put your name anywhere on it.

This survey is being conducted by
Professor Cathy Mathews
South African Medical Research Council
And
Professor Seth Kalichman
of the University of Connecticut in the United States

The information in this
survey will only be seen by the researchers.

Please answer all questions honestly.
Try not to skip any questions. You may stop doing the survey at anytime without penalty.

Please do not put your name on this survey

Thank you for agreeing to participate in this study. To remind you, this is a research study about contraception/family planning. These words are used to talk about ways to prevent pregnancy and to control when to have children. We want to learn more about what women know so that we can better help you and other women make decisions about family planning.

PLEASE TELL US ABOUT YOURSELF BY MARKING OR TICKING YOUR ANSWER OR WRITING YOUR RESPONSE IN THE SPACE PROVIDED…

A1. What is your age?

_______________ years old

A2. Are you currently employed?

Yes	No

A3. What is your highest level of education?

	No formal education
	Attended primary school but did not complete
	Completed primary school
	Attended high school but did not complete
	Completed high school
	Attended university/technikon/college but did not graduate
	Graduated from university/technikon/college

A4. Do you have a main sexual partner (fasti)?

Yes	No

A5. Do you have any casual sexual partners (khwapheni)?

Yes	No

A6. Are you currently married?

Yes	No

A7. Are you living with your sexual partner or husband?

I do not have a sexual partner or husband	Yes	No

A8. Are you currently pregnant?

Yes	No	Unsure

A9. How many pregnancies have you had in total? (This includes miscarriages, abortions and still born children.)

	None
	One
	Two
	More than two

A12. When was your youngest child born?

	I do not have a child
	2016
	2015
	2014
	2013 or before

A13. How old were you when you had your first pregnancy?

I have not been pregnant	________________ years old

A15. When in the future do you think you would you like to give birth to a child /another child?

	I do not want a child/another child
	During the next 12 Months
	12 - 24 months
	25 – 36 months
	More than 36 months
	Unsure

A16. Does your main partner or husband want a child /another child?

	I do not have a main partner or husband
	Yes
	No
	Unsure

A17. Why are you at the clinic today? (Tick all that apply.)

	To bring my baby for a checkup
	To get family planning
	For TB treatment
	For HIV treatment (antiretrovirals/ARVs)
	To get an HIV test
	To get treatment for an STI
	Other reasons (please specify): ______________________________

A18. Are you HIV positive?

	Yes
	No
	Don’t know
	Refuse to answer

NOW, PLEASE TELL US ABOUT THE FAMILY PLANNING INFORMATION AND SERVICES YOU'VE RECEIVED AT CLINICS OR DAY HOSPITALS…

B1. Have you **ever** visited a clinic for family planning?

Yes	No

B2. In the **past year**, has a health worker/nurse talked to you about whether you want to have a child in the future?

Yes	No	Unsure

B3. In the **past year**, has a health worker /nursetold you about family planning methods that you can use to avoid pregnancy?

Yes	No	Unsure

B4. In the **past year**, did a health worker/nurse recommend that you use condoms along with another family planning method?

Yes	No	Unsure

THESE QUESTIONS ARE ABOUT USING FAMILY PLANNING. WE'RE INTERESTED IN UNDERSTANDING A BIT MORE ABOUT WHAT YOU AND YOUR PARTNER THINK ABOUT FAMILY PLANNING, AND HOW YOU USE IT. WE ARE INTERESTED IN YOUR OPINION. THERE ARE NO RIGHT OR WRONG ANSWERS.

C1. Who do you think should be responsible for family planning?

The woman	Her male partner	Both the woman and her male partner

C2. Who do you think should decide whether a woman uses family planning?

The woman	Her male partner	Both the woman and her male partner

C3. When did you last have sex?

	I have never had sex
	In the past week
	In the past month
	In the past six months
	More than six months ago

C4. Have you ever used any method of family planning?

I have never had sex	Yes	No

C5. How does **your partner** feel about you using family planning?

	I do not have a partner
	I do not use family planning
	I do not know how he feels
	My partner supports my use
	My partner does not know I use family planning
	My partner does not like me to use family planning

C9. Have you ever given **your main partner** male condoms to use when you are having sex to prevent pregnancy?

I have never had a main partner	Yes	No

BELOW ARE A FEW QUESTIONS ABOUT DIFFERENT TYPES OF FAMILY PLANNING METHODS. PLEASE ANSWER AS FULLY AS POSSIBLE.

Implant:

F1. Have you heard about the **implant** (a small plastic rod inserted under the skin of a woman's arm to prevent pregnancy)?

Yes	No

F2. Where have you heard most of what you know about the implant?

	I have not heard about it
	Health worker
	Friends
	Media (radio, newspapers, the internet)
	Other (please specify): ______________________________

F3. Have you ever had an **implant**?

Yes	No

F4. Do you currently have an **implant**?

Yes	No

F5. Did you have an **implant** when you last had sex?

I have never had sex	Yes	No

F6. Has a health worker/nurse ever offered you the **implant**?	Yes	No

F7. Do you think you might use the **implant** in the future?	Yes	No	Unsure

Pill (oral contraceptive pill):

D1. Have you heard about family planning **pills**?	Yes	No

D2. Have you **ever** used family planning **pills**?	Yes	No

D3. Were you using family planning **pills** the last time you had sex?	I have never had sex	Yes	No

D4. Has a health worker ever offered you family planning **pills**?	Yes	No

D5. Do you think you might use family planning **pills** in the future?	Yes	No	Unsure

IUD:

E1. Have you heard about the intra-uterine device or **IUD** (an IUD is a loop or a coil inserted into a woman's uterus, by a health worker, to prevent pregnancy)?	Yes	No

E2. Have you **ever** had an **IUD**?

Yes	No

E3. Do you **currently** have an **IUD**?

Yes	No

E4. Did you have an **IUD** when you last had sex?

I have never had sex	Yes	No

E5. Has a health worker/nurse **ever** offered you the **IUD**?

Yes	No

E6. Do you think you might use the **IUD** in the **future**?

Yes	No	Unsure

Injection:

G1. Have you heard about the **injection** (an injection that a health worker can give a woman to stop her from becoming pregnant)?

Yes	No

G2. Have you ever used the **injection**?

Yes	No

G3. Do you currently use the **injection**?

Yes	No

G4. Were you using the **injection** when you last had sex?

I have never had sex	Yes	No

G5. Has a health worker/nurse ever offered you the **injection**?

Yes	No

G6. Do you think you might use the **injection** in the future?

Yes	No	Unsure

Emergency contraception:

H1. Have you heard about **emergency contraception** (a pill that can be taken within 3 days after sex to prevent pregnancy)?

I have never had sex	Yes	No

H2. Have you ever used **emergency contraception**?

I have never had sex	Yes	No

Male condom:

I1. Has a partner **ever** used a male **condom** during sex with you?

I have never had sex	Yes	No

I2. Did your partner use a male **condom** the **last time** you had sex?

I have never had sex	Yes	No

Female condom:

J1. Have you **ever** used a **female condom** during sex?

I have never had sex	Yes	No

J2. Did you use a **female condom** the last time you had sex?

I have never had sex	Yes	No

NOW, WE'D LIKE TO KNOW WHAT YOU THINK ABOUT THE IMPLANT. (THE IMPLANT IS A SMALL PLASTIC ROD INSERTED UNDER THE SKIN OF A WOMAN'S ARM TO PREVENT PREGNANCY). THERE ARE NO RIGHT OR WRONG ANSWERS.

K1. Most women can safely use the implant.

Agree	Disagree	Unsure

K2. Teenagers can safely use the implant.

Agree	Disagree	Unsure

K3. Women who have not yet had a baby can safely use the implant.

Agree	Disagree	Unsure

K4. Women with HIV can safely use the implant.

Agree	Disagree	Unsure

K5. The implant is very effective at preventing pregnancy.

Agree	Disagree	Unsure

	A few months

K6. The implant can prevent pregnancy for

	About 1 year
	About 3 years
	About 5 years
	More than 5 years
	Unsure

K7. The implant protects against STIs and HIV.

Agree	Disagree	Unsure

FINALLY, WE'D LIKE TO KNOW WHAT IT WOULD BE LIKE FOR YOU TO USE THE IMPLANT. IF YOU HAVE NOT USED THE IMPLANT, PLEASE IMAGINE WHAT IT WOULD BE LIKE WHEN ANSWERING THE FOLLOWING QUESTIONS…

*L1. I think that if I were to use the **implant**…*

- It could be easily inserted

Agree	Disagree	Unsure

*L2. I think that if I were to use the **implant**…*

- It could be easily removed when I wanted it to be

Agree	Disagree	Unsure

*L3. I think that if I were to use the **implant**…*

- It would be very convenient (I will not have to go to the clinic as often so it will save me time)

Agree	Disagree	Unsure

*L4. I think that if I were to use the **implant**…*

- It would be very good because it lasts for a long time

Agree	Disagree	Unsure

*L5. I think that if I were to use the **implant**…*

- I would worry about gaining weight

Agree	Disagree	Unsure

*L6. I think that if I were to use the **implant**…*

- I would be worried that my monthly bleeding would change or become irregular

Agree	Disagree	Unsure

*L7. I think that if I were to use the **implant**…*

- I could get pregnant very soon after having it removed, if I wanted to

Agree	Disagree	Unsure

*L8. I think that if I were to use the **implant**…*

- It could stop me from getting pregnant or make it harder to get pregnant in the future, even after it's taken out

Agree	Disagree	Unsure

*L9. I think that if I were to use the **implant**…*

- The implant could move around my body

Agree	Disagree	Unsure

*L10. I think that if I were to use the **implant**…*

- It would be painful

Agree	Disagree	Unsure

*L11. I think that if I were to use the **implant**…*

- It could harm babies that I have in the future

Agree	Disagree	Unsure

L23. Overall, would you say your opinion about the implant is good or bad?

Good	Bad	I have no opinion about the implant

L24. Would you like to write anything else about the implant? For example, if you think there are good or bad things about this method of family planning, or any questions you might like to ask?

____

____

____
